# Sociodemographic, Behavioral, Health, and Health Care Access Factors Associated with Frequent Mental Distress Among Non-Hispanic Multiracial U.S. Adults: A 2023 BRFSS with Policy Implications

**DOI:** 10.3390/healthcare14213664

**Published:** 2026-10-28

**Authors:** Sharmistha Roy, Ashis Kumar Biswas, Manoj Sharma

**Affiliations:** 1 Department of Social and Behavioral Health, School of Public Health, University of Nevada, Las Vegas, NV 89119, USA; 2 Department of Management Information Systems, Lee Business School, University of Nevada, Las Vegas, NV 89154, USA; biswaa1@unlv.nevada.edu; 3 Department of Internal Medicine, Kirk Kerkorian School of Medicine at UNLV, University of Nevada, Las Vegas, NV 89102, USA

**Keywords:** frequent mental distress, multiracial adults, mental health, BRFSS, health care access, physical activity, employment status

## Abstract

**Highlights:**

**What are the main findings?**

• Frequent mental distress affected approximately one in four non-Hispanic multiracial adults in the primary analytic sample.
• Cost-related barriers to medical care showed the strongest association with frequent mental distress; at the same time, unemployment, inability to work, physical inactivity, smoking, and underweight status were also associated with higher odds.

**What are the implications of the main findings?**

• Improving health care affordability and addressing employment and behavioral factors may help reduce the burden of mental distress among multiracial adults.
• The findings support prevention-oriented public health strategies that integrate mental health with health care access, healthy behaviors, and socioeconomic conditions.

**Abstract:**

**Background/Objectives**: Frequent mental distress (FMD) is an important population mental health indicator, yet non-Hispanic multiracial adults remain understudied. This study estimated FMD prevalence and examined associated sociodemographic, health care access, behavioral, and health-related factors. **Methods**: Data from the 2023 Behavioral Risk Factor Surveillance System (BRFSS) were subjected to cross-sectional analysis, accounting for strata, survey weights, and primary sampling units. FMD was defined as ≥14 mentally unhealthy days during the past 30 days. Survey-weighted logistic regression estimated adjusted odds ratios (AORs) and 95% confidence intervals (CIs). Analyses used R version 4.6.1. **Results**: Among 9899 respondents with FMD data, weighted FMD prevalence was 24.49% (95% CI 22.64–26.34%). The complete-case analysis included 7444 of 10,125 eligible respondents (73.52%). Higher odds of FMD were associated with cost-related barriers to medical care (AOR 4.49, 95% CI 3.35–6.00, *p* < 0.001), inability to work (AOR 2.38, 95% CI 1.62–3.49, *p* < 0.001), current smoking (AOR 1.88, 95% CI 1.37–2.58, *p* < 0.001), unemployment, physical inactivity, former smoking, and underweight status. Adults aged ≥65 years had lower odds than those aged 18–34 years (AOR 0.15, 95% CI 0.09–0.26, *p* < 0.001). Sensitivity analyses using a larger sample without household income (N = 8609) and prevalence-ratio regression produced broadly consistent principal findings. **Conclusions**: Approximately one-quarter of non-Hispanic multiracial adults experienced FMD. Findings identify health care affordability, employment circumstances, smoking, physical inactivity, and health-related factors as important correlates and support further research addressing mental health needs among multiracial adults.

## 1. Introduction

Mental health is an integral component of overall health and is associated with daily functioning, social relationships, employment, health behaviors, and quality of life [1]. Population-based surveillance of mental health is therefore important for identifying groups experiencing a greater burden of poor mental health and informing public health planning and prevention [2]. FMD is a population-level measure used by the Centers for Disease Control and Prevention (CDC) to monitor mental health. FMD is defined as reporting 14 or more days during the previous 30 days when mental health, including stress, depression, and problems with emotions, was not good [3]. Recent national BRFSS surveillance illustrates the magnitude of this burden. In 2022, 16.0% of U.S. adults reported FMD [4].

Mental distress is not distributed uniformly across the U.S. adult population. Social and economic circumstances, employment, access to health care, and other conditions in which people live can contribute to inequalities in mental health [5]. National BRFSS research has documented substantial variation in FMD across population groups. Cree et al. (2020) [6], for example, identified marked differences in FMD according to disability status and disability type among U.S. adults. More recent BRFSS research found that loneliness and lack of social and emotional support were associated with stress, FMD, and a history of depression [4]. These findings support examining mental distress within the social, economic, and health circumstances of specific populations rather than relying solely on overall population estimates.

The public health importance of understanding the health of multiracial populations has increased alongside substantial demographic change in the United States. The number of people identifying with two or more races increased from 9.0 million in 2010 to 33.8 million in 2020, representing a 276% increase [7]. The Census Bureau has noted that this change reflects both demographic change and improvements in the design, processing, and coding of race and ethnicity questions [7]. This growing population warrants specific attention because multiracial adults have historically been underrepresented in some areas of health research, and their experiences may not be adequately characterized when they are combined with other racial and ethnic groups [8,9].

Available evidence indicates that multiracial adults experience a range of mental health concerns. In a mixed-methods study of multiracial and multiethnic adults in the United States, Shaff et al. (2024) [9] documented depression, anxiety, posttraumatic stress, and suicidal thoughts and behaviors and identified potentially traumatic experiences and discrimination as important correlates of mental health. Franco et al. (2021) [10] further found that multiple dimensions of discrimination, including general racial discrimination, discrimination from family, and racial identity invalidation, were associated with poorer mental health among multiracial adults. A systematic review by Oh et al. (2024) [8] also demonstrated the breadth and heterogeneity of the literature on mental health outcomes among multiracial populations. Together, these findings indicate that the mental health experiences of multiracial adults warrant examination as a distinct area of population health research.

Socioeconomic circumstances are also relevant to mental health. Healthy People 2030 organizes social determinants of health into five domains, including economic stability, education access and quality, and health care access and quality [11]. Employment is included within the Economic Stability domain, and employment conditions such as job security, financial compensation, work environment, and job demands may influence health. Unemployment has also been associated with depression, anxiety, worry, low self-esteem, and other adverse health outcomes [11]. These relationships are particularly relevant when examining mental distress because employment and household income may reflect differences in financial security and access to resources, while poor mental health may itself interfere with employment and occupational functioning [5,12].

Health care access represents another important dimension of the social context in which mental distress occurs. Financial barriers to health care remain a population health concern. Healthy People 2030 reported that 8.2% of people in the United States were unable to obtain or delayed obtaining necessary medical care because of cost in 2024, compared with a national target of 5.9% [13]. The underlying data are derived from the National Health Interview Survey conducted by the CDC’s National Center for Health Statistics (ODPHP, n.d.-a) [13]. Cost-related barriers may restrict opportunities to obtain needed health services, supporting the importance of examining affordability of medical care alongside other indicators of health care access.

Health behaviors and health-related characteristics may provide additional context for understanding FMD. Physical activity has been associated with psychological well-being (Liu et al., 2024) [14], and an overview of 97 systematic reviews found that physical activity interventions were associated with improvements in depression, anxiety, and psychological distress across diverse adult populations [15]. Smoking and alcohol-related behaviors have also been examined in previous BRFSS research involving FMD (Grenard et al., 2020) [16]. Body mass index represents another relevant health-related characteristic, with evidence demonstrating associations between body mass index and mental health outcomes over time [17]. Examining these characteristics alongside socioeconomic and health care access factors may provide a broader understanding of the circumstances associated with FMD.

### 1.1. Gaps in Existing Research

Despite growing attention to racial and ethnic differences in mental health, important gaps remain in understanding FMD among non-Hispanic multiracial adults. Existing research has provided valuable evidence concerning depression, anxiety, posttraumatic stress, suicidal thoughts and behaviors, discrimination, racial identity, and related psychosocial experiences among multiracial populations [8,9,10]. However, the available literature encompasses heterogeneous populations, definitions, exposures, and mental health outcomes [8]. Consequently, findings from the overall U.S. adult population or from individual racial and ethnic groups cannot necessarily be assumed to characterize the mental health experiences of adults who identify as multiracial.

An important gap concerns FMD as a population-level indicator of sustained mental distress. Although FMD is routinely used in public health surveillance and affects a substantial proportion of U.S. adults, population-based evidence specifically characterizing FMD among non-Hispanic multiracial adults remains limited [4,6]. Much of the existing literature involving multiracial adults has instead focused on specific psychiatric symptoms, discrimination, identity-related experiences, and other psychosocial factors [8,9,10]. These studies provide an important foundation, but they do not fully address how FMD is distributed within non-Hispanic multiracial adults or which routinely measured social, behavioral, and health-related characteristics are associated with FMD in this population.

A further gap concerns the range of factors considered simultaneously. Social determinants research demonstrates that mental health is associated with economic, educational, employment, and health care circumstances [5]. Healthy People 2030 similarly identifies economic stability, education access and quality, and health care access and quality as major domains of the social determinants of health [11]. At the same time, physical activity, smoking, alcohol-related behaviors, and body mass index have been associated with mental health or examined in relation to FMD in previous research [15,16,17]. Less is known about how these socioeconomic, health care, behavioral, and health-related characteristics are associated with FMD when considered together specifically among non-Hispanic multiracial adults.

Addressing this gap is increasingly relevant given the substantial growth of the U.S. multiracial population [7]. A population-based analysis focused specifically on non-Hispanic multiracial adults can complement previous research on discrimination, racial identity, and psychosocial experiences by examining factors routinely measured through public health surveillance. The selection and organization of study variables were conceptually informed by the social determinants of health perspective described by Healthy People 2030 [11], together with prior literature on factors associated with mental health and FMD [5,15,16,17]. Accordingly, the analysis considered sociodemographic, socioeconomic, health care access, behavioral, and health-related factors. Such an analysis can provide population-specific estimates of FMD and identify characteristics associated with greater or lower odds of FMD, while providing evidence that may inform future research and population-level prevention efforts.

### 1.2. Study Objectives

The present study used data from the 2023 BRFSS to examine FMD among non-Hispanic multiracial adults in the United States. The study had two primary objectives. The first was to estimate the survey-weighted prevalence of FMD in this population. The second was to examine associations between sociodemographic characteristics, socioeconomic circumstances, health care access, health behaviors, health-related characteristics, and FMD. Because BRFSS data are cross-sectional, the findings were interpreted as associations rather than causal relationships.

## 2. Materials and Methods

### 2.1. Data Source and Study Design

This study used a cross-sectional design to analyze publicly available data from the 2023 BRFSS [18]. The BRFSS is a state-based health surveillance system coordinated by the CDC in collaboration with state and territorial health departments. The survey collects information on health status, chronic health conditions, health-related behaviors, health care access, and use of preventive health services among noninstitutionalized adults aged 18 years or older. Data are collected through landline and cellular telephone interviews using probability-based sampling procedures [19,20]. The present analysis used the 2023 annual BRFSS dataset and therefore represents information collected during the 2023 BRFSS survey year. BRFSS measures are based primarily on respondents’ self-reported information obtained during telephone interviews.

The publicly available 2023 BRFSS dataset included responses collected through landline and mobile telephone interviews across 48 U.S. states, the District of Columbia, the U.S. Virgin Islands, Puerto Rico and Guam. Kentucky and Pennsylvania did not collect sufficient data to meet the minimum requirements for inclusion in the 2023 public dataset. The final public-use dataset contained 433,323 records [19,20]. The BRFSS uses weighting procedures to account for unequal probabilities of selection, nonresponse, and differences between the sample and the population from which respondents were selected. Beginning in 2011, BRFSS incorporated cellular telephone respondents and adopted iterative proportional fitting, commonly referred to as raking, as part of its weighting methodology [20].

### 2.2. Study Population and Analytic Sample

The study population consisted of adults classified as non-Hispanic multiracial in the 2023 BRFSS. Race and ethnicity were determined using the BRFSS-calculated race and ethnicity variable (_RACE) [19]. Respondents coded as 7 on _RACE, corresponding to multiracial and non-Hispanic, were included in the study population. A total of 10,125 respondents met the race and ethnicity criterion. Among these respondents, 9899 had non-missing information for FMD, while 226 respondents had missing information for the mental health outcome.

A complete-case approach was used for the primary regression analyses. Respondents were required to have non-missing information for FMD and all covariates included in the primary multivariable model. These variables included sex, age, educational attainment, marital status, employment status, household income, cost-related barriers to medical care, physical activity, smoking status, body mass index (BMI), and alcohol use. The resulting primary analytic sample included 7444 respondents.

Because household income had a relatively large amount of missing information, a sensitivity analysis was conducted in which household income was removed from both the complete-case requirement and the multivariable model. This increased the sensitivity analysis sample to 8609 respondents.

### 2.3. Study Measures

Variable definitions and coding were based on the 2023 BRFSS questionnaire, calculated variables documentation, and codebook [19]. The original BRFSS variable names are reported in parentheses to improve reproducibility.

#### 2.3.1. Frequent Mental Distress

The primary outcome was FMD, which was derived from the BRFSS calculated mental health variable (_MENT14D) [19]. This variable is derived from the core BRFSS question asking respondents to report the number of days during the previous 30 days when their mental health was not good. The question defines mental health broadly to include stress, depression, and problems with emotions. The calculated variable (_MENT14D) classifies respondents into three categories: zero mentally unhealthy days, 1 to 13 mentally unhealthy days, and 14 or more mentally unhealthy days. Responses categorized as “do not know”, refused, or missing are assigned to the missing category. Consistent with the CDC/BRFSS definition, 14 or more mentally unhealthy days during the previous 30 days was used to define FMD [19].

For this study, a binary outcome was created. Respondents reporting 14 or more mentally unhealthy days were classified as having FMD, whereas those reporting zero to 13 mentally unhealthy days were classified as not having FMD. Responses classified as missing, do not know, or refused were treated as missing.

#### 2.3.2. Sociodemographic Characteristics

Sex was measured using (SEXVAR) and categorized as male or female. Male was used as the reference category in the regression analyses. Age was obtained from the calculated variable (_AGEG5YR). The original age categories were collapsed into four groups: 18 to 34 years, 35 to 49 years, 50 to 64 years, and 65 years or older. Respondents aged 18 to 34 years served as the reference category. Educational attainment was derived from (_EDUCAG) and categorized as college graduate, some college, high school graduate, and less than high school. College graduates served as the reference category [19].

Marital status was based on (MARITAL). Respondents who were married or members of an unmarried couple were combined into the married or partnered category. Respondents who were divorced, widowed, or separated were combined into the previously married category. Respondents who had never married formed the third category. Married or partnered respondents served as the reference category [19].

Employment status was derived from (EMPLOY1). Respondents who were employed for wages or self-employed were categorized as employed. Respondents who reported being out of work were categorized as unemployed. Students and homemakers were combined into one category. Retired respondents and respondents who were unable to work were retained as separate categories. Employed respondents served as the reference category. Annual household income was based on the calculated variable (_INCOMG1). Income was grouped into four categories: less than $25,000, $25,000 to less than $50,000, $50,000 to less than $100,000, and $100,000 or more. The $100,000 or more category served as the reference category [19].

#### 2.3.3. Health Care Access

Cost-related barriers to medical care were measured using (MEDCOST1). Respondents were asked whether there had been a time during the previous 12 months when they needed to see a doctor but could not because of cost. Responses were categorized as yes or no. Respondents who did not report a cost-related barrier served as the reference category. Responses coded as do not know, not sure, refused, or missing were treated as missing when applicable [19].

#### 2.3.4. Behavioral and Health-Related Characteristics

Physical activity was assessed using (EXERANY2), which asks whether respondents participated in any physical activity or exercise during the previous month other than their regular job. Responses were categorized as yes or no. Respondents reporting physical activity served as the reference category [19].

Smoking status was obtained from the calculated variable (_SMOKER3). Respondents classified as current everyday smokers and current some-day smokers were combined into a current smoker category. Former smokers were retained as a separate category, and respondents who had never smoked were classified as never smokers. Never smokers served as the reference category [19].

BMI was categorized using the BRFSS calculated variable (_BMI5CAT). Respondents were classified as underweight, normal weight, overweight, or obese. Normal weight served as the reference category. Alcohol use was measured using (DRNKANY6), which identifies whether respondents consumed at least one alcoholic beverage during the previous 30 days. Alcohol use was categorized as yes or no. Respondents reporting no alcohol use during the previous 30 days served as the reference category [19].

### 2.4. Missing Data Management

Values identified in the BRFSS codebook as “do not know,” “not sure,” “refused,” or missing were treated as missing, as appropriate for each study variable [19]. Variable-specific missingness among the 10,125 eligible non-Hispanic multiracial respondents is reported in Supplementary Table S1. The primary multivariable analysis used complete-case analysis, requiring nonmissing information for FMD and all covariates included in the model. This resulted in a primary analytic sample of 7444 respondents (73.52% of the eligible sample), with 2681 respondents (26.48%) excluded because of missing information on one or more analysis variables. The participant flow from the full BRFSS dataset to the primary and sensitivity analytic samples is presented in Supplementary Figure S1. Household income had the greatest amount of missing data, with 1765 respondents (17.43%) missing income information.

To assess the robustness of the findings to the reduced analytic sample and model specification, a sensitivity analysis was conducted in which household income was omitted from both the complete-case requirement and the multivariable model. This increased the analytic sample to 8609 respondents (85.03% of the eligible sample). Because this sensitivity analysis simultaneously changed the analytic sample and the covariate adjustment set, it was interpreted as an assessment of consistency across analytic samples and model specifications rather than as an isolated assessment of the effect of income missingness.

### 2.5. Statistical Analysis

All data management and statistical analyses were conducted using R version 4.6.1 (R Core Team, 2026) [21]. The analytic workflow included data import, variable recoding and derivation, management of missing responses, construction of analytic samples, specification of the BRFSS complex survey design, estimation of survey-weighted descriptive statistics and prevalence estimates, survey-weighted regression modeling, sensitivity analysis, and graphical presentation of results. The haven package (version 2.5.5) was used to import the BRFSS SAS transport file, the survey package (version 4.5) was used for complex survey analyses, and ggplot2 (version 4.0.3) was used to construct graphical displays.

The BRFSS complex sampling design was incorporated using the final sampling weight (_LLCPWT), stratification variable (_STSTR), and primary sampling unit (_PSU) (CDC, 2024) [20]. The survey design object was first constructed using the full 2023 BRFSS dataset, with primary sampling units specified as nested within strata, and was subsequently subset to respondents classified as non-Hispanic multiracial. The primary complete-case analytic sample was then obtained by restricting the target-population survey design to respondents with nonmissing information for FMD and all covariates included in the primary model. Singleton primary sampling units were handled using survey.lonely.psu = “adjust”. Variance estimation used Taylor series linearization as implemented in the survey package.

Descriptive analyses included unweighted frequencies and survey-weighted percentages. The overall survey-weighted prevalence of FMD and corresponding 95% confidence interval (CI) were estimated among respondents with non-missing FMD information (N = 9899). Survey-weighted prevalence estimates of FMD across categories of sociodemographic, socioeconomic, health care access, behavioral, and health-related characteristics were calculated within the primary complete-case analytic sample (N = 7444).

A multivariable survey-weighted logistic regression model was subsequently fitted with the prespecified covariates entered simultaneously. The unadjusted analyses used the same complete-case analytic sample as the primary adjusted model to ensure comparability across models. Regression models were fitted using the quasibinomial family within the survey-weighted generalized linear modeling framework. Results are reported as odds ratios (ORs) and adjusted odds ratios (AORs), with corresponding 95% CIs and *p*-values. Statistical tests were two-sided, and *p* < 0.05 was considered statistically significant.

Covariates were selected a priori based on their conceptual relevance to FMD and evidence from prior literature and represented sociodemographic, socioeconomic, health care access, behavioral, and health-related domains. All covariates were entered simultaneously into the multivariable model to estimate mutually adjusted associations. Multicollinearity among covariates was assessed using generalized variance inflation factors (GVIFs), with GVIF values adjusted for the degrees of freedom of categorical predictors with multiple categories. Adjusted GVIF values ranged from 1.05 to 1.24, indicating no evidence of problematic multicollinearity. To evaluate the robustness of the primary findings to missing household income information, a sensitivity analysis was conducted by removing household income from both the complete-case requirement and the multivariable model. This increased the analytic sample from 7444 to 8609 respondents. Estimates from the sensitivity model were compared with those from the primary model with respect to the direction and magnitude of the principal associations.

### 2.6. Sensitivity Analysis

A sensitivity analysis was performed to assess the consistency of the primary findings across an alternative analytic sample and model specification. Household income was omitted from both the complete-case requirement and the multivariable regression model, increasing the analytic sample from 7444 respondents in the primary analysis to 8609 respondents in the sensitivity analysis. The sensitivity model included sex, age, educational attainment, marital status, employment status, cost-related barriers to medical care, physical activity, smoking status, BMI, and alcohol use. Adjusted odds ratios and 95% CIs from the sensitivity analysis were compared with corresponding estimates from the primary model. Because omitting household income simultaneously changed the analytic sample and the covariate adjustment set, the sensitivity analysis was used to evaluate the consistency of the direction and magnitude of the principal associations across analytic samples and model specifications rather than to isolate the influence of income-related missingness.

Because FMD was relatively common in the study population, an additional sensitivity analysis was conducted to estimate adjusted prevalence ratios (APRs). A survey-weighted generalized linear model with a log link and quasipoisson family was fitted using the primary complete-case analytic sample (N = 7444) and the same covariates included in the primary logistic regression model. The model incorporated the BRFSS sampling weights, strata, and primary sampling units, with design-based standard errors used to calculate 95% CIs. APRs were obtained by exponentiating the model coefficients. Complete results from this analysis are presented in Supplementary Table S2.

### 2.7. Presentation of Results

Descriptive findings are presented as unweighted sample counts, survey-weighted percentages, and weighted FMD prevalence estimates with 95% CIs. Regression findings are presented as ORs and AORs with 95% CIs and *p*-values. The adjusted associations from the primary multivariable model are also displayed in a forest plot. Weighted FMD prevalence estimates across selected participant characteristics are presented graphically with corresponding 95% CIs. Because the BRFSS has a cross-sectional design, temporal ordering cannot be established. Therefore, regression estimates were interpreted as associations rather than causal effects or prospective predictors.

### 2.8. Reporting Guidelines

The reporting of this cross-sectional study followed the Strengthening the Reporting of Observational Studies in Epidemiology (STROBE) guidelines to support transparent and complete reporting of the study methods and findings [22].

## 3. Results

### 3.1. Study Population and Prevalence of Frequent Mental Distress

A total of 10,125 respondents in the 2023 BRFSS were classified as non-Hispanic multiracial adults and met the eligibility criteria for the present study. Among these respondents, 9899 had nonmissing information on FMD and contributed to the overall prevalence analysis. The survey-weighted prevalence of FMD among these respondents was 24.49% (95% CI 22.64–26.34%). After respondents with missing information on variables included in the primary multivariable model were excluded, the complete-case analytic sample consisted of 7444 adults. Within the primary complete-case analytic sample, the survey-weighted prevalence of FMD was 24.82% (95% CI 22.67–26.97%). Table 1 presents the unweighted sample distribution, weighted population distribution, and weighted prevalence of FMD across characteristics of the primary complete-case analytic sample.

### 3.2. Characteristics of the Analytic Sample and FMD Prevalence

The weighted distribution by sex was approximately equal, with women representing 50.49% and men representing 49.51% of the analytic population. Adults aged 18 to 34 years constituted the largest weighted age group at 40.94%, followed by adults aged 35 to 49 years at 27.53%. Approximately 36.28% had some college education, while 29.95% were college graduates. Nearly half of the weighted analytic population was married or partnered at 47.38%, and 64.11% were employed. Substantial differences in weighted FMD prevalence were observed across several characteristics. FMD prevalence was progressively lower across older age groups, ranging from 32.3% among adults aged 18 to 34 years to 12.5% among adults aged 65 years or older. FMD prevalence was 40.7% among adults unable to work and 37.4% among unemployed adults, compared with 24.0% among employed adults.

**Figure 1:**
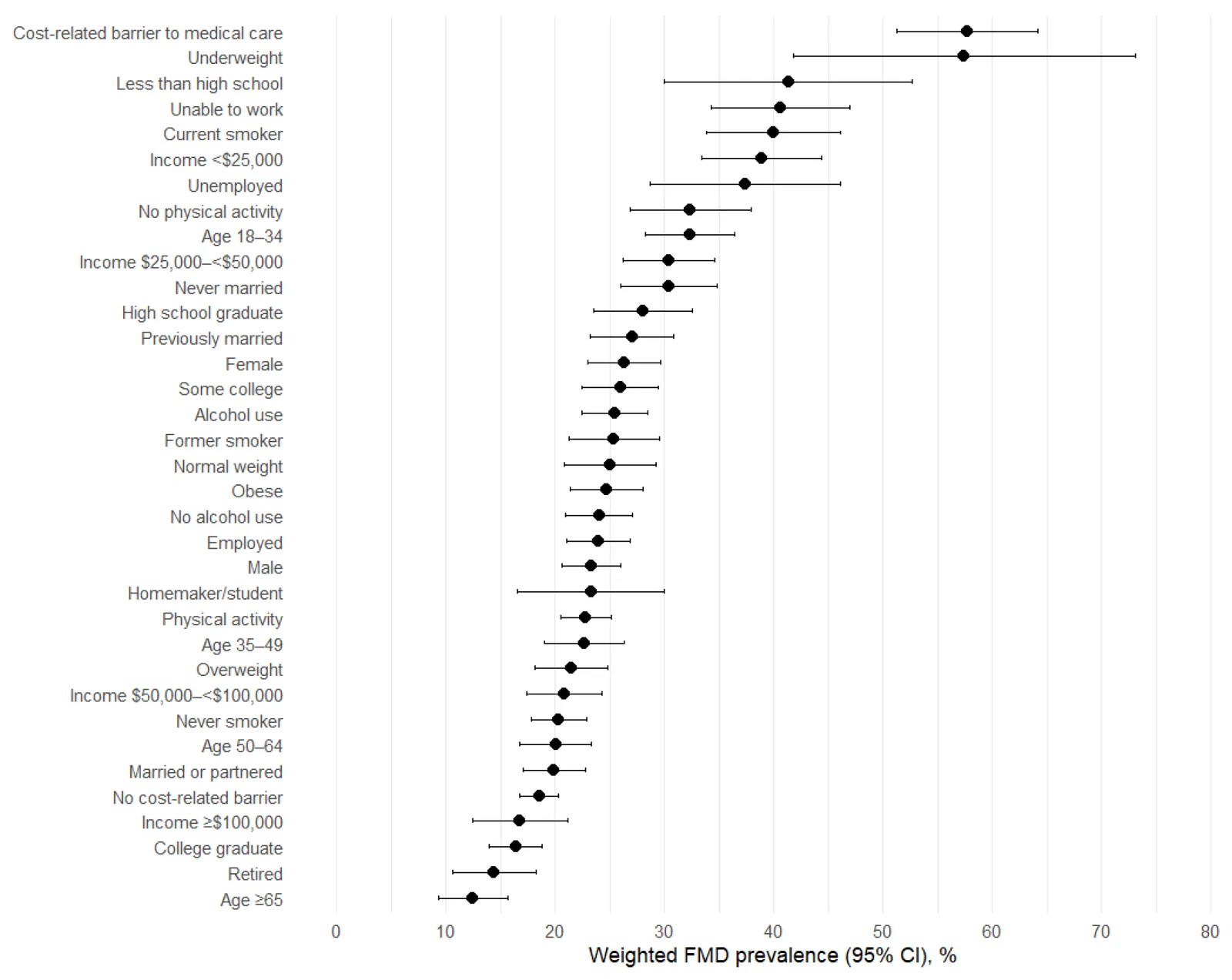
Survey-Weighted Prevalence of Frequent Mental Distress Across Selected Characteristics Among Non-Hispanic Multiracial Adults, BRFSS 2023. Note. Points represent survey-weighted FMD prevalence estimates, and horizontal lines represent 95% confidence intervals. Estimates were calculated using the primary complete-case analytic sample (N = 7444) and accounted for the BRFSS complex survey design using the final sampling weight, stratification variable, and primary sampling unit.

A particularly large descriptive difference was observed according to cost-related barriers to medical care. FMD prevalence was 57.7% among adults who reported being unable to see a doctor because of cost, compared with 18.6% among those who did not report this barrier. FMD prevalence was also higher among adults reporting no physical activity than among physically active adults, at 32.4% and 22.8%, respectively. Current smokers had an FMD prevalence of 40.0%, compared with 20.4% among never-smokers. Among BMI categories, the weighted prevalence was highest among underweight adults at 57.4%. The confidence interval for the underweight group was comparatively wide, reflecting lower precision for this estimate. Weighted FMD prevalence estimates and corresponding 95% confidence intervals across participant characteristics are presented in Figure 1.

### 3.3. Unadjusted Associations with Frequent Mental Distress

Table 2 presents the results of the unadjusted and adjusted survey-weighted logistic regression analyses. Several sociodemographic, health care access, behavioral, and health-related characteristics were significantly associated with FMD in the unadjusted analyses.

Compared with adults aged 18 to 34 years, adults aged 35 to 49 years had lower odds of FMD (OR 0.61, 95% CI 0.46 to 0.81, *p* < 0.001). Lower odds were also observed among adults aged 50 to 64 years (OR 0.53, 95% CI 0.40 to 0.69, *p* < 0.001) and adults aged 65 years or older (OR 0.30, 95% CI 0.21 to 0.42, *p* < 0.001).

Lower educational attainment was associated with higher odds of FMD before adjustment. Compared with college graduates, adults with some college education had higher odds of FMD (OR 1.79, 95% CI 1.40 to 2.30, *p* < 0.001), as did high school graduates (OR 1.99, 95% CI 1.48 to 2.67, *p* < 0.001) and adults with less than a high school education (OR 3.60, 95% CI 2.18 to 5.93, *p* < 0.001). Previously married adults (OR 1.49, 95% CI 1.14 to 1.95, *p* = 0.003) and never married adults (OR 1.76, 95% CI 1.34 to 2.30, *p* < 0.001) also had higher odds of FMD than married or partnered adults.

Compared with employed adults, unemployed adults had higher odds of FMD (OR 1.89, 95% CI 1.27 to 2.83, *p* = 0.002), as did adults unable to work (OR 2.18, 95% CI 1.60 to 2.95, *p* < 0.001). Retired adults had lower unadjusted odds of FMD (OR 0.54, 95% CI 0.38 to 0.76, *p* < 0.001).

Household income was also associated with FMD before adjustment. Compared with adults reporting annual household incomes of $100,000 or more, those with incomes below $25,000 had higher odds of FMD (OR 3.15, 95% CI 2.13 to 4.66, *p* < 0.001), as did those with incomes from $25,000 to less than $50,000 (OR 2.17, 95% CI 1.50 to 3.13, *p* < 0.001).

Cost-related barriers to medical care showed the strongest unadjusted association. Adults who reported being unable to see a doctor because of cost had nearly six times the odds of FMD compared with adults without this barrier (OR 5.99, 95% CI 4.50 to 7.99, *p* < 0.001).

Physical inactivity (OR 1.62, 95% CI 1.22 to 2.15, *p* < 0.001), current smoking (OR 2.60, 95% CI 1.93 to 3.52, *p* < 0.001), former smoking (OR 1.33, 95% CI 1.01 to 1.75, *p* = 0.039), and underweight status (OR 4.03, 95% CI 2.05 to 7.94, *p* < 0.001) were also associated with higher odds of FMD in the unadjusted analyses. Alcohol use was not significantly associated with FMD.

### 3.4. Adjusted Associations with Frequent Mental Distress

After simultaneous adjustment for all study covariates, several associations remained statistically significant. Age showed a consistent inverse association with FMD. Compared with adults aged 18 to 34 years, adults aged 35 to 49 years had lower adjusted odds of FMD (AOR 0.51, 95% CI 0.37–0.71, *p* < 0.001). Lower adjusted odds were also observed among adults aged 50 to 64 years (AOR 0.34, 95% CI 0.24–0.48, *p* < 0.001) and adults aged 65 years or older (AOR 0.15, 95% CI 0.09–0.26, *p* < 0.001).

Employment status remained associated with FMD after adjustment. Unemployed adults had higher adjusted odds of FMD than employed adults (AOR 1.57, 95% CI 1.05–2.35, *p* = 0.027). Adults who were unable to work had more than twice the adjusted odds of FMD (AOR 2.38, 95% CI 1.62–3.49, *p* < 0.001).

Cost-related barriers to medical care showed the largest adjusted association in the primary model. Adults who reported that they needed to see a doctor but could not because of cost had substantially higher adjusted odds of FMD compared with adults who did not report this barrier (AOR 4.49, 95% CI 3.35–6.00, *p* < 0.001). Adults reporting no physical activity had higher adjusted odds of FMD than physically active adults (AOR 1.46, 95% CI 1.12–1.91, *p* = 0.006). Current smokers had higher adjusted odds of FMD than never smokers (AOR 1.88, 95% CI 1.37–2.58, *p* < 0.001), as did former smokers (AOR 1.44, 95% CI 1.09–1.90, *p* = 0.011).

**Figure 2:**
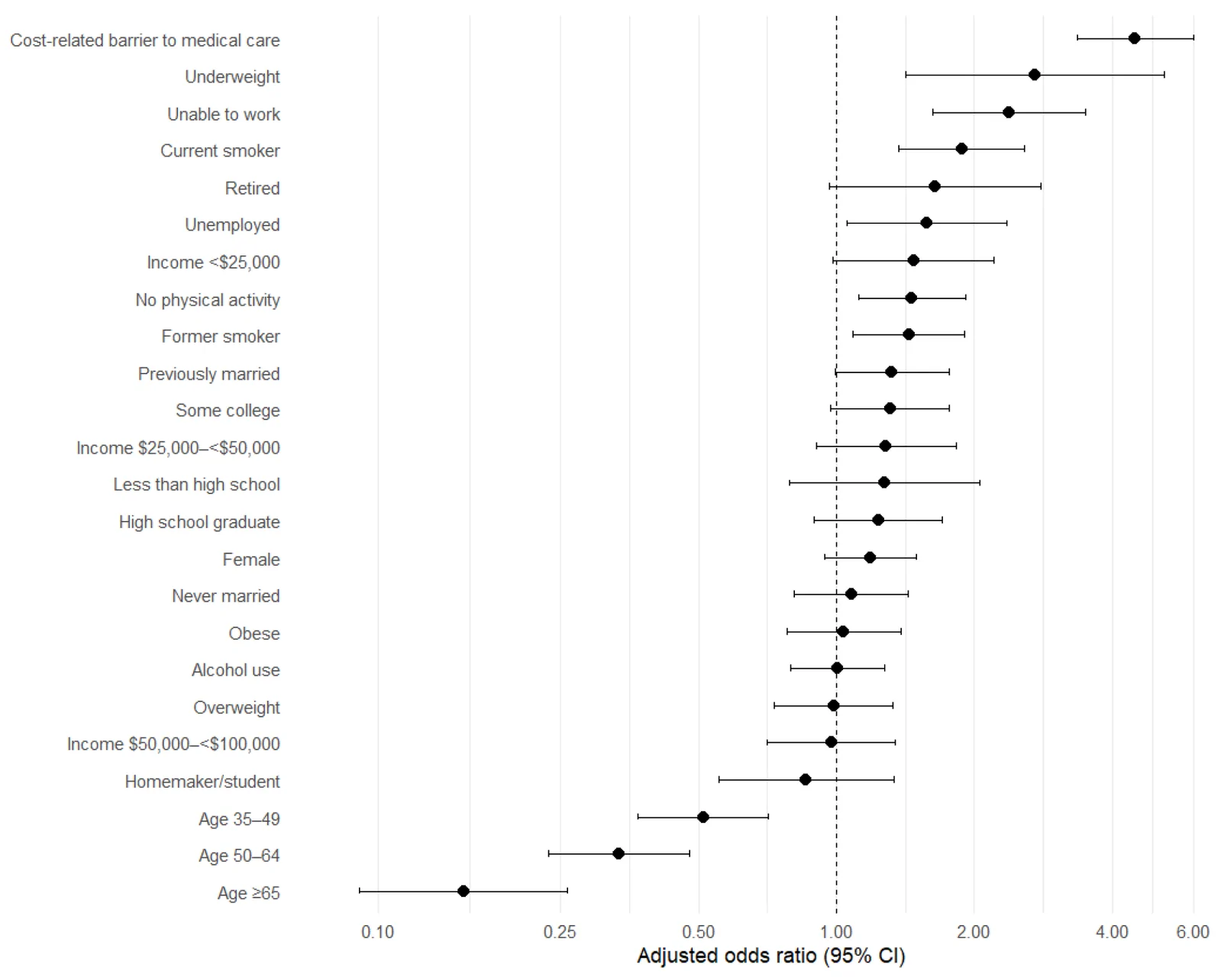
Adjusted Odds Ratios for Factors Associated with Frequent Mental Distress. Note. Points indicate adjusted odds ratios (AORs), with horizontal lines representing 95% confidence intervals. The vertical dashed line at 1.00 indicates no association. Estimates were derived from the survey-weighted multivariable logistic regression model using the primary complete-case sample (N = 7444). Reference categories are not displayed.

Underweight adults had higher adjusted odds of FMD compared with adults with normal weight (AOR 2.71, 95% CI 1.41–5.20, *p* = 0.003). The confidence interval was comparatively wide, indicating lower precision for this estimate. Overweight and obesity were not significantly associated with FMD after adjustment. Sex, educational attainment, marital status, household income, overweight status, obesity, and alcohol use were not statistically significantly associated with FMD in the fully adjusted model. The adjusted associations from the primary multivariable model are presented graphically in Figure 2.

Multicollinearity assessment showed adjusted GVIF values ranging from 1.05 to 1.24, indicating no evidence of problematic multicollinearity among the covariates included in the adjusted model.

### 3.5. Changes Between Unadjusted and Adjusted Associations

Several associations observed in the unadjusted analyses were attenuated after adjustment. Compared with college graduates, adults with less than a high school education had substantially higher odds of FMD in the unadjusted model (OR 3.60, 95% CI 2.18–5.93, *p* < 0.001), but this association was no longer statistically significant after adjustment (AOR 1.28, 95% CI 0.79–2.06, *p* = 0.316).

A similar pattern was observed for household income. Adults with annual household incomes below $25,000 had higher unadjusted odds of FMD compared with adults with incomes of $100,000 or more (OR 3.15, 95% CI 2.13–4.66, *p* < 0.001). After adjustment, the magnitude of this association was reduced and was no longer statistically significant (AOR 1.47, 95% CI 0.98–2.21, *p* = 0.061).

Associations for marital status were also attenuated after adjustment. Previously married adults had higher odds of FMD in the unadjusted analysis (OR 1.49, 95% CI 1.14–1.95, *p* = 0.003), whereas the adjusted association did not reach statistical significance (AOR 1.32, 95% CI 0.99–1.76, *p* = 0.055). The association for never married adults was also no longer significant after adjustment.

Retirement showed a change in the direction of the estimated association. Retired adults had lower odds of FMD in the unadjusted model (OR 0.54, 95% CI 0.38–0.76, *p* < 0.001). In the adjusted model, the estimated association was reversed in direction but was not statistically significant (AOR 1.64, 95% CI 0.97–2.79, *p* = 0.067).

In contrast, associations involving older age, unemployment, inability to work, cost-related barriers to medical care, physical inactivity, current smoking, former smoking, and underweight status remained statistically significant after adjustment.

### 3.6. Sensitivity Analysis

A sensitivity analysis was conducted to assess the consistency of the primary findings across an alternative analytic sample and model specification. After household income was removed from both the complete-case requirement and the multivariable model, the analytic sample increased from 7444 to 8609 respondents. Results from the primary and sensitivity analyses are compared in Table 3. The principal findings were generally consistent with those of the primary model. Older age remained associated with lower odds of FMD, while unemployment, inability to work, cost-related barriers to medical care, physical inactivity, current smoking, former smoking, and underweight status remained associated with higher odds of FMD.

In the sensitivity analysis, unemployed adults had higher odds of FMD (AOR 1.51, 95% CI 1.05–2.17, *p* = 0.026), as did adults unable to work (AOR 2.35, 95% CI 1.68–3.28, *p* < 0.001). Cost-related barriers to medical care remained strongly associated with FMD (AOR 4.11, 95% CI 3.15–5.36, *p* < 0.001). Higher odds of FMD were also observed for physical inactivity (AOR 1.46, 95% CI 1.14–1.88, *p* = 0.003), current smoking (AOR 2.12, 95% CI 1.59–2.83, *p* < 0.001), former smoking (AOR 1.44, 95% CI 1.11–1.88, *p* = 0.007), and underweight status (AOR 2.15, 95% CI 1.15–4.05, *p* = 0.017).

Several secondary associations differed between the primary and sensitivity models. Female sex (AOR 1.32, 95% CI 1.07–1.64, *p* = 0.011), some college education (AOR 1.43, 95% CI 1.09–1.87, *p* = 0.010), high school education (AOR 1.42, 95% CI 1.03–1.94, *p* = 0.030), and previously married status (AOR 1.40, 95% CI 1.07–1.82, *p* = 0.014) were statistically significant in the sensitivity model, although these associations were not statistically significant in the primary model. Overall, the direction and magnitude of the principal associations were generally similar across the two analyses. Because household income was omitted from both the complete-case requirement and the multivariable model, the sensitivity analysis simultaneously changed the analytic sample and the covariate adjustment set. Therefore, the influence of income-related missingness cannot be distinguished from the influence of the modified model specification.

An additional sensitivity analysis was conducted using survey-weighted prevalence-ratio regression because FMD was relatively common in the study population. In this analysis, cost-related barriers to medical care showed the strongest positive association with FMD (APR 2.34, 95% CI 2.01–2.72, *p* < 0.001). Higher FMD prevalence was also observed among adults who were unable to work (APR 1.69, 95% CI 1.36–2.09, *p* < 0.001), physically inactive (APR 1.26, 95% CI 1.08–1.46, *p* = 0.003), current smokers (APR 1.39, 95% CI 1.16–1.67, *p* < 0.001), former smokers (APR 1.23, 95% CI 1.03–1.47, *p* = 0.023), and underweight (APR 1.54, 95% CI 1.18–2.01, *p* = 0.001). Unemployed adults also had higher FMD prevalence (APR 1.30, 95% CI 1.04–1.61, *p* = 0.019). Adults aged 35–49 years (APR 0.64, 95% CI 0.52–0.79, *p* < 0.001), 50–64 years (APR 0.46, 95% CI 0.36–0.59, *p* < 0.001), and ≥65 years (APR 0.34, 95% CI 0.23–0.52, *p* < 0.001) had lower FMD prevalence than adults aged 18–34 years. Complete results from the prevalence-ratio sensitivity analysis are presented in Supplementary Table S2.

## 4. Discussion

### 4.1. Principal Findings

This study examined FMD among non-Hispanic multiracial adults using data from the 2023 BRFSS. Approximately one in four adults with nonmissing FMD information experienced FMD, with a survey-weighted prevalence of 24.49% (95% CI 22.64–26.34%). After adjustment for sociodemographic, socioeconomic, health care access, behavioral, and health-related characteristics, several factors remained significantly associated with FMD. The largest adjusted association was observed for cost-related barriers to medical care. Unemployment, inability to work, physical inactivity, current and former smoking, and underweight status were also associated with higher odds of FMD. In contrast, older age was consistently associated with substantially lower odds of FMD compared with adults aged 18 to 34 years (Table 2).

Previous population-based research provides additional context for these findings. Using 2019 BRFSS data, Rashid et al. (2022) [23] reported that approximately 12% of U.S. adults experienced FMD and identified multiracial identity, younger age, lower income, and underweight status among characteristics associated with greater mental distress. Similarly, Liao et al. (2022) [24], using BRFSS data from 2013 to 2017, documented associations between mental distress and several chronic health conditions, highlighting the importance of considering physical health alongside psychological well-being. Although these studies examined broader adult populations and differed in their outcome measures or analytic approaches, they provide relevant context for the present investigation.

These findings add to a growing but still limited literature on mental health among multiracial adults. Previous studies have documented depression, anxiety, posttraumatic stress, suicidal thoughts and behaviors, discrimination, and identity-related stressors among multiracial populations [8,9,10].

Shaff et al. (2024) [9] identified potentially traumatic experiences and discrimination as important correlates of mental health among multiracial and multiethnic adults, while Franco et al. (2021) [10] found that general racial discrimination, discrimination from family, and racial identity invalidation were associated with poorer mental health among multiracial adults. Oh et al. (2024) [8], in a systematic review, further demonstrated that mental health outcomes among multiracial individuals are heterogeneous and shaped by individual and social experiences. The present study extends this literature by focusing specifically on FMD and examining multiple routinely measured social, health care access, behavioral, and health-related characteristics in a population-based sample of non-Hispanic multiracial adults. These principal associations were generally supported by prevalence-ratio sensitivity analysis.

### 4.2. Cost-Related Barriers to Medical Care and Frequent Mental Distress

Cost-related barriers to medical care showed the largest adjusted association with FMD. Adults who reported that they needed to see a doctor but could not because of cost had more than four times the adjusted odds of FMD compared with those who did not report this barrier. The descriptive findings showed a similarly pronounced difference, with weighted FMD prevalence of 57.7% among adults reporting a cost-related barrier compared with 18.6% among those without such a barrier (Table 1).

This finding is consistent with the broader social determinants of mental health literature. Kirkbride et al. (2024) [5] synthesized evidence demonstrating that socioeconomic circumstances and access to resources and services are closely related to mental health and mental disorders. Healthy People 2030 likewise identifies health care access and quality as a major social determinant of health and monitors the proportion of people unable to obtain or delay necessary medical care because of cost [13]. Previous population-based studies have also documented associations between mental health status and unmet health care needs. Jones (2021) [25], using nationally representative U.S. data, found that adults with poorer self-rated mental health had greater odds of reporting unmet medical care needs. Similarly, Johnson et al. (2022) [26] reported that midlife adults experiencing severe psychological distress, particularly those with multiple chronic conditions, had substantially higher odds of foregoing necessary health care because of cost. The present findings extend this evidence by demonstrating a particularly large association between cost-related inability to obtain medical care and FMD among non-Hispanic multiracial adults.

Several pathways may contribute to this association. Difficulty affording medical care may coexist with financial strain, inadequate insurance coverage, competing household expenses, or unmet physical and mental health needs. However, the relationship may also operate in the opposite direction. Individuals experiencing persistent mental distress may face employment difficulties, reduced financial resources, or increased health care needs that make affordability barriers more likely. Because the BRFSS is cross-sectional, the present analysis cannot determine the temporal direction of the relationship. Nevertheless, the magnitude of the association suggests that health care affordability is an important consideration when examining mental distress in this population.

### 4.3. Employment Status and Frequent Mental Distress

Employment status was also strongly associated with FMD. Compared with employed adults, unemployed respondents had significantly higher adjusted odds of FMD, while respondents who were unable to work had more than twice the adjusted odds. These findings are consistent with evidence demonstrating relationships between employment circumstances and mental health. Kirkbride et al. (2024) [5] identified employment and economic conditions among the social determinants that can shape mental health across the life course. Healthy People 2030 similarly places employment within the Economic Stability domain and recognizes that unemployment is associated with adverse health and mental health outcomes [11].

The particularly elevated odds among adults unable to work are also consistent with previous BRFSS evidence concerning disability and mental distress. Cree et al. (2020) [6] found substantial disparities in FMD according to disability status and disability type among U.S. adults. Although being unable to work should not be considered equivalent to disability, the comparison provides relevant context because inability to work may coexist with chronic disease, disability, functional limitations, or other health problems. The present findings extend previous population-level evidence by showing that inability to work remained associated with FMD after simultaneous adjustment for demographic, socioeconomic, health care access, behavioral, and health-related characteristics.

Retirement showed a different pattern. Retired adults had lower odds of FMD in the unadjusted analysis, but the association changed direction and was no longer statistically significant after adjustment. This attenuation suggests that the unadjusted relationship may have reflected differences in age or other characteristics between retired and employed adults rather than an independent association between retirement and FMD.

### 4.4. Age Differences in Frequent Mental Distress

Age demonstrated one of the most consistent patterns in the study. Compared with adults aged 18 to 34 years, adults in every older age group had substantially lower adjusted odds of FMD. Weighted FMD prevalence decreased across older age groups, ranging from 32.3% among adults aged 18 to 34 years to 12.5% among adults aged 65 years or older (Table 1). This age gradient is consistent with literature showing that mental health and exposure to social and economic stressors vary across the life course [5]. Similarly, Zullo et al. (2022) [27], using BRFSS data from 2014 to 2018, reported that FMD generally decreased with increasing age among sexual and gender minority adults and their heterosexual, cisgender counterparts. Although their study examined a different population, it provides additional population-based evidence of age-related differences in FMD. The present findings are particularly noteworthy because age differences remained substantial after adjustment for education, marital status, employment, household income, health care affordability, physical activity, smoking, BMI, and alcohol use. Thus, the observed age pattern was not fully accounted for by these measured characteristics.

The mechanisms underlying this pattern cannot be established from the present data. Younger adults may differ from older adults in employment conditions, economic security, social roles, exposure to stressors, coping resources, or willingness to report psychological distress. Multiracial adults may additionally experience age- or life-stage-specific issues involving racial identity, discrimination, belonging, and identity invalidation. Previous research has demonstrated the relevance of discrimination and racial identity experiences to mental health among multiracial populations [9,10]. However, these mechanisms were not directly measured and should be examined in future research.

### 4.5. Physical Activity and Frequent Mental Distress

Adults who reported no physical activity outside of their regular job had significantly higher adjusted odds of FMD than physically active adults. This finding is consistent with previous evidence linking physical activity with mental health. Singh et al. (2023) [15], in an overview of 97 systematic reviews, found that physical activity interventions were associated with improvements in depression, anxiety, and psychological distress across diverse adult populations. Liu et al. (2024) [14] similarly reported relationships among physical activity, health behavior, mental health, and psychological resilience. Rashid et al. (2022) [23] also examined lifestyle characteristics in relation to FMD using nationally representative BRFSS data, providing additional epidemiological context for the observed association.

The present study extends this evidence by demonstrating that the association between physical inactivity and mental distress remained significant after adjustment for sociodemographic, socioeconomic, health care access, behavioral, and health-related characteristics among non-Hispanic multiracial adults. However, the association may be bidirectional. Physical activity may support psychological well-being, while individuals experiencing substantial mental distress may have reduced motivation, energy, social engagement, or opportunities for physical activity. In addition, the BRFSS measure assessed whether respondents engaged in any physical activity or exercise outside of their regular job during the previous month and did not capture detailed information on frequency, intensity, or duration. Therefore, the findings should be interpreted as evidence of an association rather than a causal effect of physical inactivity on FMD.

### 4.6. Smoking Status and Frequent Mental Distress

Smoking status was associated with FMD after adjustment. Current smokers had significantly higher adjusted odds of FMD than never smokers, and former smokers also had elevated adjusted odds, although the magnitude of the association was smaller. Weighted FMD prevalence was approximately 40.0% among current smokers compared with 20.4% among never smokers (Table 1).

These findings are consistent with previous BRFSS research demonstrating connections between smoking behavior and mental distress. Grenard et al. (2020) [16] examined FMD together with smoking and drinking behaviors in a population-based U.S. context, supporting the relevance of considering tobacco use when examining mental distress. More broadly, smoking and mental health have a complex relationship involving psychological, behavioral, socioeconomic, and environmental factors.

The present findings add to this literature by showing that current smoking remained associated with FMD after adjustment for socioeconomic circumstances, health care access, physical activity, BMI, alcohol use, and other characteristics among non-Hispanic multiracial adults. However, the cross-sectional design prevents determination of temporal direction. Smoking may be used by some individuals in response to psychological distress, while nicotine dependence and withdrawal may also be associated with psychological symptoms. Shared social and economic circumstances may contribute to both behaviors. Accordingly, the findings should not be interpreted as demonstrating that smoking causes FMD. The elevated odds among former smokers also warrant caution because this group may differ in duration and intensity of previous smoking, time since cessation, reasons for quitting, and underlying health conditions. These factors were not examined in the present analysis.

### 4.7. Body Mass Index and Frequent Mental Distress

Underweight adults had significantly higher adjusted odds of FMD compared with adults with normal weight. The weighted prevalence of FMD among underweight adults was 57.4%, the highest observed among the BMI categories (Table 1). However, only 148 respondents in the complete-case analytic sample were classified as underweight, and the corresponding adjusted estimate had a comparatively wide confidence interval. Therefore, this finding should be interpreted cautiously.

The finding is relevant to previous evidence demonstrating that the relationship between BMI and mental health is complex and may vary across the BMI distribution. Chen et al. (2024) [17] reported dose-dependent associations between BMI and mental health and demonstrated that relationships between body weight and mental health may change over time. The present finding contributes to the literature by identifying a potentially important association at the lower end of the BMI distribution among non-Hispanic multiracial adults.

Several explanations are possible, including nutritional status, underlying chronic disease, socioeconomic circumstances, appetite changes accompanying psychological distress, or other unmeasured health conditions. Reverse causation is also possible because psychological distress may affect appetite, eating behavior, and body weight. These potential mechanisms could not be evaluated in the present study.

In contrast, overweight and obesity were not significantly associated with FMD after adjustment. This does not demonstrate that excess body weight is unrelated to mental health. Rather, after accounting for the other measured characteristics in this population, overweight and obesity did not show statistically significant associations with FMD. This pattern supports the possibility that the relationship between BMI and mental health is heterogeneous rather than simply linear [17].

### 4.8. Socioeconomic and Sociodemographic Factors Attenuated After Adjustment

Several characteristics associated with FMD in the unadjusted analyses were attenuated after simultaneous adjustment. Educational attainment and household income showed particularly clear patterns. Adults with lower educational attainment and lower household income had higher odds of FMD in the unadjusted analyses, but these associations were no longer statistically significant in the fully adjusted model (Table 2).

These results should be considered in the context of evidence linking socioeconomic circumstances with mental health. Kirkbride et al. (2024) [5] identified economic and educational circumstances as important social determinants of mental health and disorder. Healthy People 2030 recognizes employment as an important component of economic stability and a social determinant of health [11]. Rashid et al. (2022) [23], using nationally representative BRFSS data, also identified socioeconomic and sociodemographic characteristics associated with FMD among U.S. adults. Therefore, the attenuation observed in the present study should not be interpreted as indicating that education or income is unimportant to mental health.

Instead, the differences between unadjusted and adjusted estimates suggest that these characteristics share statistical relationships with other factors in the model, including employment status, health care affordability, and health behaviors. For example, household income may be closely connected with employment and the ability to afford health care. Adjustment for these interrelated characteristics may therefore reduce the association attributed to income itself. Because the analysis was cross-sectional, these findings cannot determine whether any of these factors mediate or cause the observed relationships.

Marital status followed a similar pattern. Previously married and never married adults had higher unadjusted odds of FMD than married or partnered adults, but these associations were attenuated after adjustment. Bruss et al. (2024) [4] reported associations between lack of social and emotional support, loneliness, and mental health problems in BRFSS data, illustrating the potential importance of social relationships to population mental health. However, marital status is not equivalent to social support, and the present study did not directly measure relationship quality, loneliness, or perceived support. Therefore, the attenuation of marital-status associations should be interpreted cautiously.

### 4.9. Sensitivity Analysis

The sensitivity analysis showed that the principal findings were generally consistent across the alternative analytic sample and model specification. After household income was omitted from both the complete-case requirement and the multivariable model, the analytic sample increased from 7444 to 8609 respondents. The major associations involving age, unemployment, inability to work, cost-related barriers to medical care, physical inactivity, smoking status, and underweight status remained generally consistent in direction and magnitude.

However, several secondary associations differed between the models. Female sex, some college education, high school education, and previously married status were statistically significant in the sensitivity model but not in the primary model. These differences indicate that some secondary estimates were sensitive to changes in the analytic sample and model specification. Because household income was omitted from both the complete-case requirement and the multivariable model, the influence of income-related missingness cannot be separated from the influence of the changed adjustment set. Therefore, the sensitivity analysis supports the general consistency of the principal associations across the two model specifications but does not establish that missing income information had no influence on the results. Detailed comparisons between the primary and sensitivity models are presented in Table 3.

The additional prevalence-ratio sensitivity analysis further supported the principal findings while producing effect estimates that were generally smaller in magnitude than the corresponding odds ratios, as expected for a relatively common outcome. Cost-related barriers to medical care, unemployment, inability to work, physical inactivity, current and former smoking, underweight status, and younger age remained associated with FMD. These findings indicate that the principal conclusions were not dependent on expressing the associations as odds ratios. Complete results from the prevalence-ratio analysis are presented in Supplementary Table S2.

### 4.10. Public Health Implications

The findings have implications for public health research, practice, and national health priorities. The strong association between cost-related barriers to medical care and FMD highlights the importance of considering mental health within the broader context of health care affordability. Healthy People 2030 identifies reducing cost-related barriers to necessary medical care as a national objective (ODPHP, 2024) [13]. The associations with unemployment and inability to work further underscore the relevance of socioeconomic circumstances, consistent with the social determinants of mental health framework described by Kirkbride et al. (2024) [5] and the Economic Stability domain of Healthy People 2030 [11].

These findings are also relevant to federal health priorities emphasizing prevention and health promotion. The Make America Healthy Again (MAHA) Commission has emphasized prevention, healthy lifestyles, and greater attention to behavioral and other potential drivers of poor health, including mental health concerns [28]. In this context, the observed associations of FMD with health care affordability, physical inactivity, smoking, and employment circumstances identify areas that may warrant further investigation in prevention-oriented public health research. However, the cross-sectional design does not establish that modifying these factors would reduce FMD. Accordingly, the findings should inform the identification of research priorities rather than be interpreted as evidence of intervention effectiveness.

The higher burden of FMD among younger adults also warrants further investigation. Future research should examine whether discrimination, racial identity invalidation, belonging, social support, and economic insecurity contribute to the observed patterns among non-Hispanic multiracial adults. Previous studies have linked discrimination and racial identity experiences with mental health in multiracial populations [8,9,10]. Such research could help inform approaches that consider mental health alongside health care access, health behaviors, and socioeconomic conditions. Future prevention and mental health promotion efforts may also benefit from culturally responsive approaches that recognize differences in racial identities and experiences rather than treating multiracial adults as a uniform population. The effectiveness of these approaches should be evaluated in future studies.

### 4.11. Strengths and Limitations

This study has several strengths. It used a large, population-based health surveillance dataset to examine an understudied population and considered a broad range of sociodemographic, health care access, behavioral, and health-related factors. Analyses accounted for the BRFSS complex survey design. The sensitivity analysis assessed the consistency of the principal findings when household income was excluded, whereas the additional prevalence-ratio analysis evaluated their robustness given the relatively high prevalence of FMD.

Several limitations should be considered. First, the cross-sectional design prevents determination of temporal sequence or causal relationships. Therefore, the identified factors should be interpreted as correlates of FMD rather than established causes or consequences. Second, BRFSS data are largely self-reported and may be subject to recall, reporting, and social desirability biases. FMD reflects self-reported mentally unhealthy days rather than a clinical psychiatric diagnosis. Potential nonresponse bias may also remain despite BRFSS weighting procedures.

Third, complete-case analysis reduced the primary analytic sample from 10,125 eligible respondents to 7444 (73.52%), potentially introducing selection bias if excluded respondents differed systematically from those retained. Household income had the greatest missingness (17.43%). Although excluding household income from the complete-case requirement increased the sensitivity sample to 8609 and yielded broadly consistent principal findings, both the analytic sample and adjustment set changed simultaneously. Consequently, this analysis cannot isolate the influence of income-related missingness from changes in model specification or fully rule out selection bias associated with missing data. The relatively small number of underweight respondents also resulted in a less precise estimate for this group.

Finally, BRFSS does not capture experiences potentially relevant to multiracial mental health, including racial identity invalidation, discrimination, and social belonging [8]. Non-Hispanic multiracial adults are also a heterogeneous population, and analyzing them as a single group may obscure differences across racial identities, racial combinations, and social experiences. Future studies with sufficiently large samples should examine these factors and heterogeneity within multiracial populations.

## 5. Conclusions

Frequent mental distress was common among non-Hispanic multiracial adults in the 2023 Behavioral Risk Factor Surveillance System, affecting approximately one in four adults with nonmissing FMD information. Cost-related barriers to medical care showed the strongest adjusted association with FMD. Unemployment, inability to work, physical inactivity, current and former smoking, and underweight status were also associated with higher odds of FMD, whereas older adults had substantially lower odds than those aged 18 to 34 years.

These findings highlight the relevance of health care affordability, employment circumstances, and behavioral and health-related factors to understanding mental distress among multiracial adults. They suggest potential priorities for further investigation, including affordable health care access, economic and functional challenges, and health behaviors. However, the cross-sectional design prevents causal interpretation, and the findings do not establish whether interventions targeting these factors would reduce FMD. Future longitudinal research should examine discrimination, racial identity invalidation, social belonging, and heterogeneity across multiracial groups. Overall, this study provides population-based evidence to inform future research and the development and evaluation of public health approaches for this understudied population.

## Figures and Tables

**Table 1:** Characteristics of the Complete-Case Analytic Sample and Weighted Prevalence of Frequent Mental Distress, N = 7444.

Characteristic	Unweighted *n*	Weighted %	Weighted FMD Prevalence, %
**Sex**			
Male	3765	49.51	23.31
Female	3679	50.49	26.30
**Age, years**			
18–34	2054	40.94	32.34
35–49	1981	27.53	22.64
50–64	1752	18.55	20.06
≥65	1657	12.97	12.51
**Educational attainment**			
College graduate	2864	29.95	16.40
Some college	2369	36.28	25.98
High school graduate	1859	26.22	28.05
Less than high school	352	7.55	41.36
**Marital status**			
Married or partnered	3610	47.38	19.93
Previously married	1809	18.80	27.07
Never married	2025	33.82	30.42
**Employment status**			
Employed	4469	64.11	23.96
Unemployed	402	5.68	37.37
Homemaker or student	541	10.94	23.28
Retired	1397	11.62	14.44
Unable to work	635	7.66	40.66
**Annual household income**			
≥$100,000	2041	30.28	16.80
<$25,000	1242	15.47	38.92
$25,000–<$50,000	1912	25.03	30.43
$50,000–<$100,000	2249	29.22	20.85
**Cost-related barrier to medical care**			
No	6489	84.04	18.56
Yes	955	15.96	57.74
**Physical activity**			
Yes	5802	79.20	22.83
No	1642	20.80	32.39
**Smoking status**			
Never smoker	4110	58.08	20.36
Current smoker	1254	16.10	39.97
Former smoker	2080	25.82	25.39
**BMI category**			
Normal weight	2159	32.66	25.07
Underweight	148	3.01	57.42
Overweight	2420	30.88	21.48
Obese	2717	33.45	24.73
**Alcohol use**			
No	3409	44.38	24.04
Yes	4035	55.62	25.44

Note. FMD = frequent mental distress. Unweighted n values represent respondents in the complete-case analytic sample. Weighted percentages and weighted FMD prevalence estimates account for the BRFSS complex survey design using the final sampling weight, stratification variable, and primary sampling unit. Percentages may not total 100.0 because of rounding.

**Table 2:** Survey-Weighted Logistic Regression of Factors Associated with FMD, N = 7444.

Characteristic	Unadjusted OR (95% CI)	*p* Value	Adjusted OR (95% CI)	*p* Value
**Sex**				
Male	Ref	—	Ref	—
Female	1.17 (0.94–1.47)	0.157	**1.19 (0.94–1.49)**	**0.143**
**Age, years**				
18–34	Ref	—	Ref	—
35–49	0.61 (0.46–0.81)	<0.001	0.51 (0.37–0.71)	<0.001
50–64	0.53 (0.40–0.69)	<0.001	**0.34 (0.24–0.48)**	<0.001
≥65	0.30 (0.21–0.42)	<0.001	0.15 (0.09–0.26)	<0.001
**Educational attainment**				
College graduate	Ref	—	Ref	—
Some college	1.79 (1.40–2.30)	<0.001	**1.31 (0.97–1.77)**	**0.079**
High school graduate	1.99 (1.48–2.67)	<0.001	**1.23 (0.90–1.70)**	**0.198**
Less than high school	3.60 (2.18–5.93)	<0.001	1.28 (0.79–2.06)	**0.316**
**Marital status**				
Married or partnered	Ref	—	Ref	—
Previously married	1.49 (1.14–1.95)	0.003	**1.32 (0.99–1.76)**	**0.055**
Never married	1.76 (1.34–2.30)	<0.001	1.08 (0.81–1.44)	**0.605**
**Employment status**				
Employed	Ref	—	Ref	—
Unemployed	1.89 (1.27–2.83)	0.002	**1.57 (1.05–2.35)**	**0.027**
Homemaker or student	0.96 (0.64–1.46)	0.859	**0.86 (0.55–1.33)**	**0.497**
Retired	0.54 (0.38–0.76)	<0.001	**1.64 (0.97–2.79)**	**0.067**
Unable to work	2.18 (1.60–2.95)	<0.001	**2.38 (1.62–3.49)**	<0.001
**Annual household income**				
≥$100,000	Ref	—	Ref	—
<$25,000	3.15 (2.13–4.66)	<0.001	1.47 (0.98–2.21)	**0.061**
$25,000–<$50,000	2.17 (1.50–3.13)	<0.001	**1.29 (0.90–1.83)**	**0.162**
$50,000–<$100,000	1.30 (0.89–1.90)	0.168	0.97 (0.71–1.35)	**0.877**
**Cost-related barrier to medical care**				
No	Ref	—	Ref	—
Yes	5.99 (4.50–7.99)	<0.001	4.49 (3.35–6.00)	<0.001
**Physical activity**				
Yes	Ref	—	Ref	—
No	1.62 (1.22–2.15)	<0.001	**1.46 (1.12–1.91)**	**0.006**
**Smoking status**				
Never smoker	Ref	—	Ref	—
Current smoker	2.60 (1.93–3.52)	<0.001	1.88 (1.37–2.58)	<0.001
Former smoker	1.33 (1.01–1.75)	0.039	**1.44 (1.09–1.90)**	**0.011**
**BMI category**				
Normal weight	Ref	—	Ref	—
Underweight	4.03 (2.05–7.94)	<0.001	2.71 (1.41–5.20)	**0.003**
Overweight	0.82 (0.61–1.10)	0.181	**0.99 (0.73–1.33)**	**0.926**
Obese	0.98 (0.74–1.31)	0.901	1.04 (0.78–1.38)	**0.799**
**Alcohol use**				
No	Ref	—	Ref	—
Yes	1.08 (0.85–1.36)	0.529	**1.00 (0.79–1.27)**	**0.969**

Note. OR = odds ratio; AOR = adjusted odds ratio; CI = confidence interval; Ref = reference category. The adjusted model included sex, age, educational attainment, marital status, employment status, annual household income, cost-related barriers to medical care, physical activity, smoking status, body mass index category, and alcohol use simultaneously. Analyses were conducted using the final sampling weight, stratification variables, and primary sampling units. Statistical significance was defined as *p* < 0.05.

**Table 3:** Sensitivity Analysis of Factors Associated with Frequent Mental Distress Among Non-Hispanic Multiracial Adults, BRFSS 2023.

Characteristic	Primary Analysis AOR (95% CI), N = 7444	*p* Value	Sensitivity Analysis AOR (95% CI), N = 8609	*p* Value
**Sex**				
Male	Ref	—	Ref	—
Female	1.19 (0.94–1.49)	0.143	1.32 (1.07–1.64)	0.011
**Age, years**				
18–34	Ref	—	Ref	—
35–49	0.51 (0.37–0.71)	<0.001	0.49 (0.36–0.67)	<0.001
50–64	0.34 (0.24–0.48)	<0.001	0.32 (0.23–0.45)	<0.001
≥65	0.15 (0.09–0.26)	<0.001	0.16 (0.10–0.26)	<0.001
**Educational attainment**				
College graduate	Ref	—	Ref	—
Some college	1.31 (0.97–1.77)	0.079	1.43 (1.09–1.87)	0.010
High school graduate	1.23 (0.90–1.70)	0.198	1.42 (1.03–1.94)	0.030
Less than high school	1.28 (0.79–2.06)	0.316	1.37 (0.88–2.13)	0.169
**Marital status**				
Married or partnered	Ref	—	Ref	—
Previously married	1.32 (0.99–1.76)	0.055	1.40 (1.07–1.82)	0.014
Never married	1.08 (0.81–1.44)	0.605	1.01 (0.74–1.36)	0.961
**Employment status**				
Employed	Ref	—	Ref	—
Unemployed	1.57 (1.05–2.35)	0.027	1.51 (1.05–2.17)	0.026
Homemaker or student	0.86 (0.55–1.33)	0.497	1.03 (0.66–1.61)	0.895
Retired	1.64 (0.97–2.79)	0.067	1.40 (0.85–2.31)	0.188
Unable to work	2.38 (1.62–3.49)	<0.001	2.35 (1.68–3.28)	<0.001
**Annual household** **income**				
≥$100,000	Ref	—	Not included	—
<$25,000	1.47 (0.98–2.21)	0.061	Not included	—
$25,000–<$50,000	1.29 (0.90–1.83)	0.162	Not included	—
$50,000–<$100,000	0.97 (0.71–1.35)	0.877	Not included	—
**Cost-related barrier** **to medical care**				
No	Ref	—	Ref	—
Yes	4.49 (3.35–6.00)	<0.001	4.11 (3.15–5.36)	<0.001
**Physical activity**				
Yes	Ref	—	Ref	—
No	1.46 (1.12–1.91)	0.006	1.46 (1.14–1.88)	0.003
**Smoking status**				
Never smoker	Ref	—	Ref	—
Current smoker	1.88 (1.37–2.58)	<0.001	2.12 (1.59–2.83)	<0.001
Former smoker	1.44 (1.09–1.90)	0.011	1.44 (1.11–1.88)	0.007
**BMI category**				
Normal weight	Ref	—	Ref	—
Underweight	2.71 (1.41–5.20)	0.003	2.15 (1.15–4.05)	0.017
Overweight	0.99 (0.73–1.33)	0.926	0.93 (0.70–1.23)	0.604
Obese	1.04 (0.78–1.38)	0.799	1.21 (0.90–1.63)	0.202
**Alcohol use**				
No	Ref	—	Ref	—
Yes	1.00 (0.79–1.27)	0.969	0.99 (0.79–1.24)	0.931

Note. AOR = adjusted odds ratio; CI = confidence interval; Ref = reference category. The primary complete-case analysis included household income and consisted of 7444 respondents. The sensitivity analysis excluded household income from the complete-case requirement and multivariable regression model, increasing the analytic sample to 8609 respondents. Both models accounted for the BRFSS complex survey design using the final sampling weight, stratification variable, and primary sampling unit. Statistical significance was defined as *p* < 0.05.

## Data Availability

The data analyzed in this study are publicly available through the U.S. Centers for Disease Control and Prevention (CDC) Behavioral Risk Factor Surveillance System (BRFSS). The analysis used the publicly accessible 2023 BRFSS dataset: https://www.cdc.gov/brfss/annual_data/annual_2023.html (accessed on 21 July 2026).

## Supplementary Materials

The following supporting information can be downloaded at: https://www.mdpi.com/article/10.3390/healthcare14213664/s1, Table S1. Variable-specific missing data among eligible non-Hispanic multiracial adults, 2023 BRFSS (N = 10,125); Table S2. Survey-Weighted Adjusted Prevalence Ratios for Frequent Mental Distress Among Non-Hispanic Multiracial Adults, 2023 BRFSS (N = 7444); Figure S1. Participant Flow for the Primary and Sensitivity Analytic Samples, 2023 BRFSS.

## Abbreviations

Abbreviation	Full Term
AOR	Adjusted odds ratio
BMI	Body mass index
CI	Confidence interval
MAHA	Make America Healthy Again
ODPHP	Office of Disease Prevention and Health Promotion
OR	Odds ratio
PSU	Primary sampling unit
