# Supplementary material for: Sociodemographic, Behavioral, Health, and Health Care Access Factors Associated with Frequent Mental Distress Among Non-Hispanic Multiracial U.S. Adults: A 2023 BRFSS with Policy Implications
Source: Healthcare (Basel). 2026 Oct 28;14(21):3664. doi: 10.3390/healthcare14213664 (PMC13763324; doi:10.3390/healthcare14213664)
Supplement: Supplementary file 1 [file healthcare-14-03664-s001.zip › healthcare-4605874-supplementary.pdf]

Supplementary Materials

Supplementary Table S1. Variable-specific missing data among eligible non-Hispanic multiracial adults (N = 10,125).

Variable	Nonmissing n	Missing n	Missing %
Frequent mental distress	9899	226	2.23
Sex	10,125	0	0.00
Age	9974	151	1.49
Educational attainment	10,102	23	0.23
Marital status	10,037	88	0.87
Employment status	9990	135	1.33
Household income	8360	1765	17.43
Cost-related barrier to medical care	10,072	53	0.52
Physical activity	10,095	30	0.30
Smoking status	9711	414	4.09
BMI category	9402	723	7.14
Alcohol use	9602	523	5.17

Note: Percentages were calculated using the 10,125 eligible non-Hispanic multiracial respondents as the denominator. Missing values include responses coded as do not know, not sure, refused, or missing, as applicable to each BRFSS variable.

Supplementary Table S2. Survey-Weighted Adjusted Prevalence Ratios for Frequent Mental Distress Among Non-Hispanic Multiracial Adults, 2023 BRFSS (N = 7444).

Predictor	APR	95% CI	*p*-value
**Sex**			
Male	Reference		
Female	1.12	0.98–1.29	0.100
**Age, years**			
18–34	Reference		
35–49	0.64	0.52–0.79	<0.001
50–64	0.46	0.36–0.59	<0.001
≥65	0.34	0.23–0.52	<0.001
**Educational attainment**			
College graduate	Reference		
Some college	1.22	1.00–1.50	0.054
High school graduate	1.19	0.96–1.47	0.116
Less than high school	1.20	0.93–1.56	0.164
**Marital status**			
Married or partnered	Reference		
Previously married	1.30	1.06–1.59	0.011
Never married	1.18	0.97–1.43	0.100
**Employment status**			
Employed	Reference		
Unemployed	1.30	1.04–1.61	0.019
Homemaker or student	0.93	0.70–1.24	0.626
Retired	1.21	0.83–1.76	0.333
Unable to work	1.69	1.36–2.09	<0.001
**Household income, USD**			
≥100,000	Reference		

<25,000	1.31	1.03–1.67	0.029
25,000–<50,000	1.23	0.98–1.55	0.075
50,000–<100,000	1.04	0.83–1.31	0.717
**Cost-related barrier to medical care**			
No	Reference		
Yes	2.34	2.01–2.72	<0.001
**Physical activity**			
Yes	Reference		
No	1.26	1.08–1.46	0.003
**Smoking status**			
Never smoker	Reference		
Former smoker	1.23	1.03–1.47	0.023
Current smoker	1.39	1.16–1.67	<0.001
**BMI category**			
Normal weight	Reference		
Underweight	1.54	1.18–2.01	0.001
Overweight	0.96	0.80–1.16	0.700
Obesity	1.01	0.85–1.21	0.899
**Alcohol use**			
No	Reference		
Yes	0.98	0.84–1.15	0.823

Note: APR = adjusted prevalence ratio; CI = confidence interval; BMI = body mass index; BRFSS = Behavioral Risk Factor Surveillance System. APRs were estimated using a survey-weighted generalized linear model with a log link and quasipoisson family, accounting for BRFSS sampling weights, strata, and primary sampling units. The model simultaneously adjusted for all variables shown in the table. Reference categories are indicated. *p*-values <0.05 were considered statistically significant.

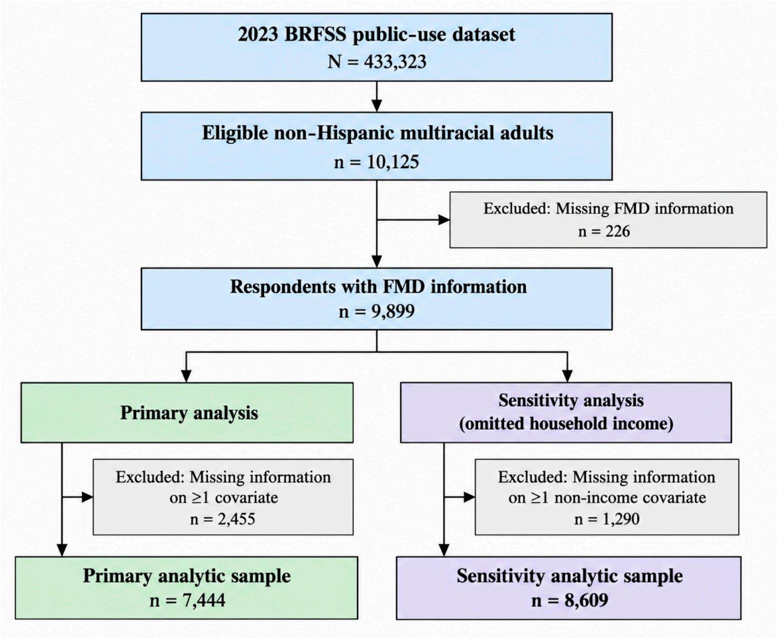

Supplementary Figure S1. Participant Flow for the Primary and Sensitivity Analytic Samples, 2023 BRFSS.
